# Genetic Diversity and Clonal Structure of Small-Leaved Lime (*Tilia cordata* Mill.) in Lithuanian Protected Forest Areas

**DOI:** 10.3390/plants15081207

**Published:** 2026-04-15

**Authors:** Rita Verbylaitė, Jūratė Lynikienė, Artūras Gedminas, Valeriia Mishcherikova, Virgilijus Baliuckas, Vytautas Suchockas

**Affiliations:** Lithuanian Research Centre for Agriculture and Forestry, Institute of Forestry, Kėdainiai District, LT-58344 Akademija, Lithuania; jurate.lynikiene@lammc.lt (J.L.); arturas.gedminas@lammc.lt (A.G.); valeriia.mishcherikova@lammc.lt (V.M.); virgilijus.baliuckas@lammc.lt (V.B.); vytautas.suchockas@lammc.lt (V.S.)

**Keywords:** populations, tree genetic monitoring, microsatellites, genetic conservation units

## Abstract

*Tilia cordata* Mill. is a long-lived, ecologically important broadleaved tree species that maintains high genetic diversity despite habitat fragmentation and historical range shifts. In this study, we assessed genetic diversity, clonal structure, and population differentiation in six genetic conservation units (GCUs) in Lithuania using nuclear microsatellite markers. A total of 1109 individuals were successfully genotyped, revealing 979 unique multi-locus genotypes, with 17% of individuals assigned to clonal lineages. Clonal groups were generally small and spatially restricted, indicating localized vegetative regeneration. Genetic diversity was high across all populations, with similar levels of observed and expected heterozygosity, consistent with predominantly outcrossing reproduction. Juvenile cohorts exhibited slightly higher allelic richness and latent genetic potential compared to mature trees, suggesting effective regeneration and maintenance of genetic variation. Genetic differentiation among populations was low but significant (*F_ST_* = 0.013; *G*″*_ST_* = 0.051), with evidence of clustering corresponding to provenance regions. High gene flow (*Nm* ≈ 10) likely contributes to weak population structure, although regional differentiation persists. The results demonstrate that Lithuanian *T. cordata* populations retain a robust genetic framework, combining high within-population diversity with moderate structuring. These findings highlight the importance of conserving multiple GCUs and implementing genetic monitoring to ensure long-term population viability under changing environmental conditions.

## 1. Introduction

*Tilia cordata* Mill. is a long-lived, deciduous broadleaved tree native to temperate and hemiboreal regions of Europe, where it occurs both as a canopy component and as a shade-tolerant subcanopy species [1]. The species exhibits high ecological plasticity and tolerates a wide range of soil types and climatic conditions. It is characterized by a mixed reproductive strategy combining sexual reproduction with vegetative propagation through coppicing and root sprouting [2,3]. These traits contribute to the long-term persistence of *T. cordata* in fragmented forest landscapes and to the survival of relict populations near the northern limits of its distribution [1,4].

*T. cordata* is a functionally important tree species contributing to ecosystem services in temperate forest systems. As a long-lived, shade-tolerant species forming both canopy and subcanopy layers, it increases structural complexity and supports biodiversity by providing habitat and resources for a wide range of organisms, including pollinators attracted to its nectar-rich flowers [5]. The species also influences soil processes, as inputs from honeydew and leaf litter enhance microbial activity and contribute to nitrogen enrichment, thereby affecting nutrient cycling [6]. In addition, *T. cordata* contributes to climate regulation through carbon sequestration and microclimate stabilization due to its dense canopy and phenological traits, such as late leaf flushing [6]. Its tolerance of environmental variation, including drought and temperature fluctuations, further supports ecosystem resilience under changing climatic conditions [7]. Moreover, *Tilia* spp. provides cultural and provisioning ecosystem services, including applications in forestry, urban environments, and apiculture [8].

Population genetic studies based on nuclear microsatellite (SSR) markers indicate that *T. cordata* maintains moderate-to-high genetic diversity within populations despite historical range contractions and habitat fragmentation [9,10,11]. Observed heterozygosity is typically close to expected heterozygosity, indicating predominantly outcrossing mating systems, although vegetative reproduction may locally increase clonality and influence genotypic structure [9,12]. Genetic differentiation among populations is generally low to moderate, suggesting effective gene flow, likely mediated by insect pollination and occasional long-distance dispersal along riparian systems [10,11].

At broader spatial scales, genetic variation in *T. cordata* reflects both postglacial recolonization processes and more recent anthropogenic influences, including forest management and landscape fragmentation [10,13,14]. These processes result in a resilient genetic framework combined with fine-scale structuring shaped by ecological gradients, reproductive biology, and land-use history.

The aim of the present study was to investigate genetic diversity and population structure in protected *T. cordata* forest stands in Lithuania and to establish a baseline for long-term genetic monitoring.

## 2. Results

For the genetic analysis of *T. cordata*, 91 individuals (76 mature trees and 15 juveniles) were excluded due to poor amplification at several loci. The total number of samples retained for each GCU is given in Table 1. A total of 14 SSR loci were used for further analysis. This number of loci was sufficient to provide reliable and reproducible estimates of genetic diversity [15]. Locus Tc31 was excluded due to a high proportion of missing data, as amplification failed in 543 samples.

Among the 1109 analyzed trees, 979 unique multi-locus genotypes (MLGs) were identified, indicating the presence of clonal groups in the studied GCUs. Of all individuals, 17% (191 trees) were assigned to clonal lineages. No identical genotypes were detected across different GCUs. In total, 62 clonal groups were identified, with 6% of MLGs represented by clones. Most clonal groups consisted of only two ramets (42 groups), typically comprising a mature tree and a nearby juvenile (23 groups).

As expected from the sampling design (where nearly all mature individuals were sampled regardless of spatial), the highest number of clonal individuals was observed in the ANK seed stand. In this GCU, 20 clonal groups were identified. Among 193 individuals analyzed in ANK, only 115 unique MLGs were detected. Clonal groups in ANK also had the highest number of ramets, with an average of five ramets, per group and a maximum of 15 ramets. Maps and the spatial distribution of clonal groups for each GCU are provided in Figure S1.

For subsequent analyses, only one representative of each MLG was retained. All but one microsatellite locus (Tc8) were polymorphic, with an average of 12.85 alleles per locus. The highest number of alleles was observed at locus Tc963 (35 alleles), while the lowest number—six alleles—was recorded for loci Tc943, Tc11 and Tc951. Detailed locus characteristics, including primer information, are presented in Table S1.

Most loci showed no evidence of null alleles (frequency < 0.1). However, loci Tc4, Tc943, Tc11 and Tc963 indicated a possible presence of null alleles. The estimated frequencies were low to moderate (Table S1) and are unlikely to substantially affect estimates of genetic differentiation among *T. cordata* populations [16]. The presence of null alleles is commonly reported in population genetic studies [16,17] and is expected in widespread tree species with large effective population sizes [16].

Genetic diversity parameters for mature and juvenile generations across populations are presented in Table 1. The number of different alleles (*Na*) ranged from 7.5 in the mature generation of ANK to 9.0 in JU2 juveniles. The effective number of alleles (*Ae*) and allelic richness (*Ar*) were generally higher in juvenile cohorts, except in JU1 and ROK genetic reserves, where mature trees exhibited higher values (JU1: *Ae* = 4.06 vs. 3.63; *Ar* = 7.29 vs. 6.54; ROK: *Ae* = 4.02 vs. 3.88; *Ar* = 6.88 vs. 6.69) (Table 1). Observed and expected heterozygosity (*Ho* and *He*) were similar across populations and between generations within populations. Differences in genetic diversity parameters (*Na*, *Ae*, *Ar*, *Ho*, *He*, number of rare and private alleles and *F_IS_*) among populations and generations were not statistically significant based on pairwise *T* tests. The number of rare alleles (frequency ≤ 0.05) was high in both generations ranging from 44 in JU1 juveniles to 74 in JU2 juveniles out of a total of 168 alleles detected (Table 1). In general, juvenile cohorts contained more rare alleles than mature cohorts, except in JU1 and ROK. The number of private alleles was relatively low, with 20 detected overall. JU2 contained the highest number (11), followed by ANK (5), while RAS had no private alleles. JU1 and ROK each had one private allele. The inbreeding coefficient (*F_IS_*) was close to zero in four of the six populations (for both generations), indicating random mating. In contrast, the ROK reserve showed a deficit of heterozygotes in both mature and juvenile cohorts (*F_IS_* = 0.145 and 0.148, respectively), and ANK showed a heterozygote deficit in the mature generation (*F_IS_* = 0.22) (Table 1). Estimated effective population sizes (*Ne*) ranged from 33.5 in the mature ANK generation to 1740.9 in the mature JU1 generation. All other populations had *Ne* values exceeding 100 in both juvenile and mature generations (Table 1). Latent genetic potential (*LGP*) was equal to or higher in juvenile cohorts in all populations except JU1, where it was lower in juveniles (*LGP* = 4.15 in mature trees vs. 3.58 in juveniles) (Table 1). The estimated number of migrants per generation among all investigated populations was high (*Nm* = 10.033 ± 1.268), indicating substantial gene flow.

**Figure 1:**
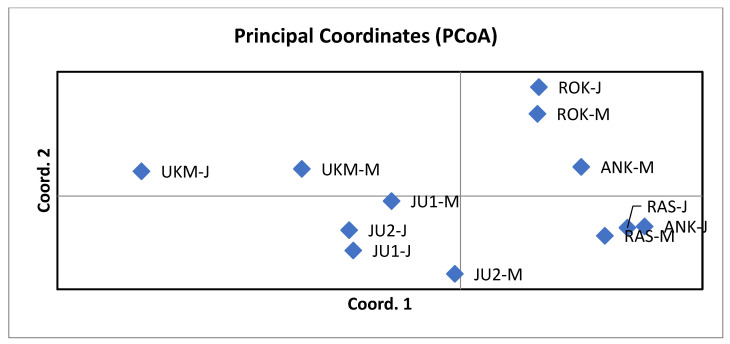
Principal coordinate analysis of mature and juvenile generations of six *Tilia cordata* Mill. populations. For genetic conservation unit (GCU) codes, please see Table 2; the letters M and J next to the GCU code designate mature and juvenile generations, respectively. The first coordinate explains 26.02% and the second 22.86% of variation.

Principal coordinate analysis (*PCoA*) revealed that the different generations of the ROK and UKM populations were more similar to each other than to those of other populations. The JU1 and JU2 populations formed a distinct group, with their juvenile cohorts positioned very close to one another. In contrast, the juvenile cohort of the ANK population was more similar to the RAS population than to its own mature cohort (Figure 1). Overall, populations from the first and third provenance regions of *T. cordata* in Lithuania (Table 2) formed separate clusters, with JU1, JU2 and RAS GCUs grouping together in the lower part of the *PCoA* plot.

**Figure 2:**
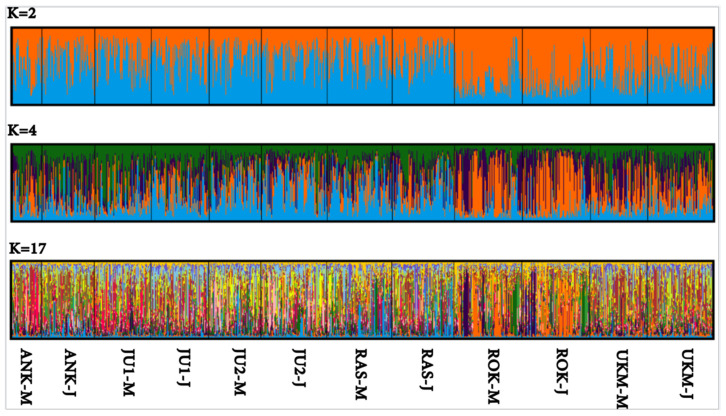
*Tilia. cordata* Mill. population genetic structure as identified by Bayesian analysis results. Each analyzed multi-locus genotype is shown as a separate vertical bar in a diagram, where a color denotes the part of the genome that belongs to each of the two clusters (K) (top histogram), four clusters (middle histogram), or 17 clusters (bottom histogram). For genetic conservation unit (GCU) codes, please see Table 2; the letters M and J next to the GCU code designate mature and juvenile generations, respectively.

Based on the results of the Bayesian clustering approach implemented in STRUCTURE software, the most probable number of clusters for *T. cordata* in Lithuanian GCUs, as determined by the *ΔK* criterion [18], was two (K = 2). The next most probable numbers of clusters were K = 4 and K = 17 (Figure 2), with the likelihood of probabilities provided in Table S2. The clustering revealed patterns similar to those observed in the *PCoA* analysis (Figure 1), with the most distinct populations being ROK and UKM.

**Figure 3:**
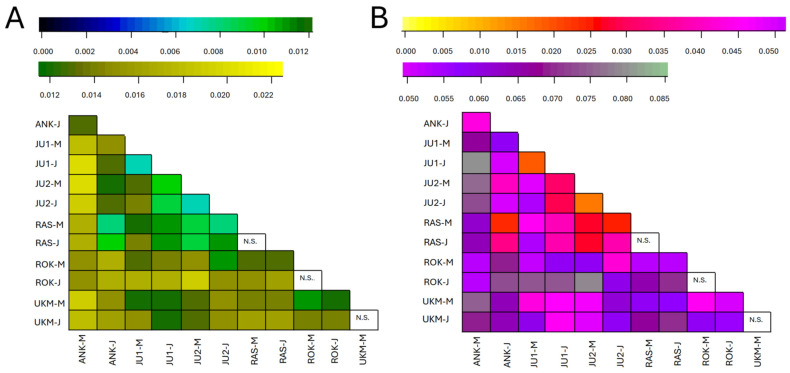
Heat map of pairwise *F_ST_* (**A**) and *G*″*_ST_* (**B**) values among all *Tilia cordata* Mill. subpopulations (mature and juvenile). N.S.—statistically not significant. For genetic conservation unit (GCU) codes, please see Table 2; the letters M and J next to the GCU code designate mature and juvenile generations, respectively.

Genetic differentiation between generations of *T. cordata* populations, as well as among populations, was low but statistically significant. Both *F_ST_* (Figure 3A) and Nei’s standardized *G*″*_ST_* (Figure 3B), which corrects for bias when the number of populations is bellow 20 (in this study six populations, or 12 groups), where applied. Although both analyses revealed a very similar patterns, *G*″*_ST_* showed a higher magnitude of differentiation (Figure 3). No significant differences between generations (i.e., between mature and juvenile cohorts within the same population) were detected in three GCUs—the RAS seed stand, and the ROK and UKM genetic reserves. Overall, genetic differentiation among populations was higher within the third provenance region GCUs (ANK, ROK and UKM; see Table 1), whereas populations from the first provenance region (JU1, JU2 and RAS; see Table 1) exhibited lower levels of differentiation among themselves.

## 3. Discussion

### 3.1. Clonality

The clonal structure observed in *T. cordata*, with 17% of individuals assigned to clonal lineages, is consistent with the species’ mixed reproductive strategy combining sexual and vegetative reproduction. Clonal propagation occurs through coppicing, root sprouting, and layering, resulting in spatially restricted groups of genetically identical ramets [2,7,9,12]. Most clonal groups consisted of two ramets, typically representing a mature tree and an adjacent juvenile individual, indicating localized vegetative regeneration rather than extensive clonal expansion [9,12].

No identical multi-locus genotypes were detected among different GCUs, indicating that clonality does not contribute to long-distance dispersal, and that gene flow occurs primarily via seed and pollen dispersal along landscape corridors [11]. The higher number and size of clonal groups in the ANK seed stand can be attributed to both ecological conditions and sampling design, as intensive sampling of mature individuals increases the probability of detecting clonal structures, particularly in stands influenced by disturbance or management [12].

The presence of clones in this study contrasts with the absence of clonality reported by Danusevičius et al. [11], which may be explained by differences in sampling design and sample size per population. More exhaustive sampling increases the likelihood of detecting neighboring ramets belonging to the same genet. The observed level of clonality is also consistent with ecological trends showing increased vegetative reproduction toward the distribution margins of *T. cordata*, particularly under suboptimal conditions that limit seed production and recruitment [19,20]. Under such conditions, vegetative propagation enhances persistence. In Latvian populations, approximately one-third of individuals were reported to be clonal, indicating that vegetative reproduction can constitute a substantial component of population structure in northern regions [7].

Despite the presence of clonal individuals, the high number of unique genotypes indicates that overall genetic diversity remains high. This supports the view that clonality contributes to local persistence without substantially reducing genetic variation [9]. Thus, clonal reproduction plays a complementary role, while sexual reproduction maintains genetic diversity and connectivity among populations [10,11].

### 3.2. Genetic Diversity and Population Differentiation

The genetic diversity patterns observed in *T. cordata* populations indicate high levels of within-population variation, as reflected by allelic richness, effective number of alleles, and heterozygosity values, which were similar among populations and generations (Table 1). In the present study, genetic diversity was high, with an average *He* = 0.62 [21]. The close correspondence between observed and expected heterozygosity suggests random mating and a predominantly outcrossing reproductive system, consistent with previous studies [7,9,10,11,12]. These characteristics are typical of long-lived, insect-pollinated tree species with high gene flow, which maintain genetic diversity despite fragmentation [1,13]. The lack of substantial differences between *Ho* and *He* provides indirect evidence of predominantly outcrossing reproduction in the investigated *T. cordata* stands. However, *F_IS_* values close to zero (a key measure comparing observed and expected heterozygosity) may also result from balancing selection or the mixing of subpopulations.

Higher allelic richness, effective allele number, and latent genetic potential in juvenile cohorts compared to mature trees in most populations indicate ongoing regeneration and effective recruitment. This suggests that genetic diversity is maintained or slightly increased across generations. Similar patterns have been reported in fragmented populations, where juvenile cohorts contribute additional rare alleles and enhance evolutionary potential [10].

Genetic differentiation among populations was low but statistically significant (Figure 3), with average values of *F_ST_* = 0.013 and *G*″*_ST_* = 0.051. These values are lower than those reported for Latvian (*F_ST_* = 0.064), UK (*F_ST_* = 0.071), and Danish (*F_ST_* = 0.034) populations [7,9,12], indicating relatively weak population structure. This pattern reflects a balance between historical isolation and ongoing gene flow [10,11]. The estimated number of migrants per generation (*Nm* ≈ 10) further supports substantial gene exchange among populations, likely facilitated by dispersal along riparian systems [11].

Nevertheless, clustering of populations according to provenance regions in both *PCoA* (Figure 1) and Bayesian analyses (Figure 2) indicates that geographic and ecological factors contribute to genetic structuring. However, AMOVA did not detect a significant proportion of genetic variation explained by provenance regions in the present study. The observed pattern is consistent with previous findings in Lithuania, where regional differentiation among populations has been reported [11]. Such structuring may be attributed to postglacial recolonization from multiple refugia and subsequent anthropogenic influences, including forest management and landscape fragmentation [10,14].

## 4. Concluding Remarks and Implications for Long-Term Conservation

The results indicate that *T. cordata* populations in Lithuania maintain high within-population genetic diversity and low-to-moderate inter-population differentiation, reflecting a resilient genetic system with substantial evolutionary potential. At the same time, the presence of a weak but significant population structure highlights the importance of conserving multiple genetic conservation units across provenance regions to preserve the full range of genetic variation [22,23].

To ensure the long-term maintenance of genetic diversity and structure, genetic monitoring should be implemented following established forest genetic monitoring frameworks [24]. We recommend assessing the genetic diversity of regenerating *T. cordata* cohorts at approximately 10-year intervals and comparing the results over time to detect potential changes. Where feasible, advanced level genetic monitoring should be applied, including seed-based genetic analyses to estimate gene flow, multi-locus outcrossing rates, effective pollen donor numbers and biparental inbreeding. In particular, increased natural regeneration should be promoted in the ANK seed stand, where higher clonality was observed. If this site is used for seed production, sampling strategies should avoid collecting material from individuals belonging to the same clone to prevent genetic redundancy.

## 5. Materials and Methods

### 5.1. Study Sites and Sampling

**Figure 4:**
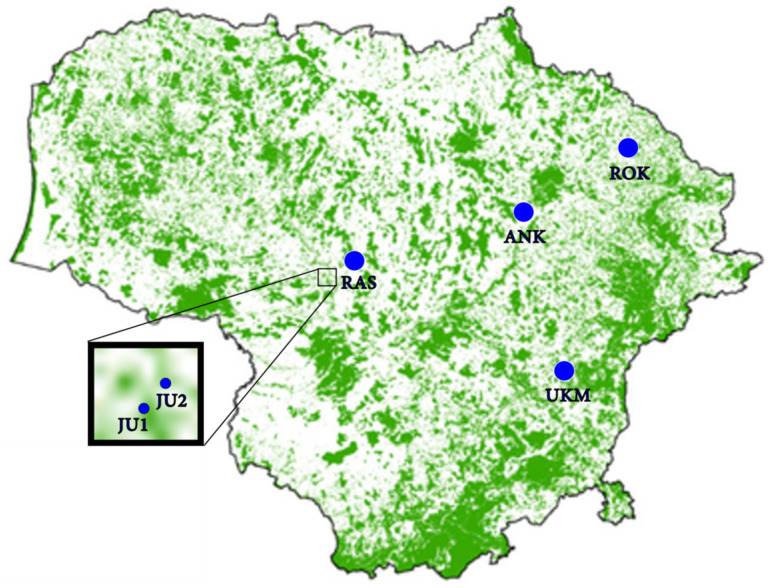
The map shows position of investigated *Tilia cordata* Mill. Genetic conservation units. The green color in the map represents forests.

In the present study, all six *T. cordata* sites designated for species conservation in Lithuania were investigated: four genetic reserves (two in the Jurbarkas regional branch of the State Forest Enterprise (SFE), codes 23LGD001 (JU1) and 23LGD002 (JU2), one in the Ukmergė regional branch of SFE, code 58LGD004 (UKM), and one in the Rokiškis regional branch of SFE, code 55LGD003 (ROK)) and two seed stands (Raseiniai regional branch of SFE, code 17LSM003 (RAS), and Anykščiai regional branch of SFE, code 46LSM002 (ANK)) (Figure 4; Table 2). All investigated *T. cordata* genetic conservation units (GCUs) were mixed-species mature forests, where the proportion of *T. cordata* in the canopy layer was of at least 40%. The age of *T. cordata* trees in the studied stands was at least 79 years, and natural regeneration (1–10-year-oldindividuals) was present (Table 2). The investigated GCUs are representative of the species’ distribution in Lithuania, as the majority of *T. cordata* dominated stands occur in the central and southern parts of the country, with approximately one-third located in the northeastern region [25].

Investigated GCUs represent two different provenance regions of *T. cordata* in Lithuania (Table 2). All sampled populations met the minimum size requirements for dynamic in situ GCUs of forest tree genetic diversity and corresponded to Case 1 (≥500 reproducing trees) [22,23].

In total, 1200 individual trees were sampled, with 200 trees per study site. At each site, half of the sampled individuals were mature trees N = 100 (canopy-forming, seed bearing individuals), while the other half comprised young regeneration N = 100 (naturally regenerated individuals aged 1–10 years, representing the progeny generation). To maximize the capture of genetic diversity and avoid sampling vegetatively regenerated individuals, the distance between sampled individuals was of at least 20 m. Juvenile individuals, where possible, were sampled in proximity (within a 5–10 m radius) to mature trees (one juvenile per mature tree, if present). The sampling distance among mature trees was not maintained in the ANK seed stand. This stand had undergone selective felling five years prior to this study to promote natural regeneration of *T. cordata*, and only a small portion of the mature stand remained. Therefore, in the ANK seed stand, nearly all remaining mature *T. cordata* trees were sampled regardless of distance. Samples from mature trees were collected using an electric drill, creating a 2–3 cm deep borehole in the trunk at a height of 1.3 m above the root collar, and collecting the resulting sawdust into 2 mL sterile tubes. Leaf samples (2–3 per tree) were collected from juvenile individuals and placed in individual paper bags. All samples were transported to the laboratory on the same day and stored at −20 °C until DNA extraction.

### 5.2. Laboratory Procedures

Before DNA extraction, 100 mg of wood or 50 mg of leaf tissue was homogenized in liquid nitrogen using a mortar and pestle. The resulting powder was immediately used for DNA extraction. DNA extraction followed the protocol described by Verbylaitė et al. [26] using the ATMAB method [27]. SSR analysis of *T. cordata* was performed as described by Phuekvilai and Wolff [13] using 14 SSR primers and primer Tc23, as described by Hansen et al. [28] (Table S1). PCR amplification was carried out in four multiplex reactions (Table S1). After amplification, PCR products were run on a 1.5% TAE agarose gel to confirm amplification success and specificity. PCR products from multiplexes A and B were pooled (mix E) using equimolar concentrations for fragment size analysis, while products from multiplexes C and D were analyzed separately. Fragment size analysis was performed by Genoscreen Innovative Genomics (Lille, France), using an ABI 3730XL DNA Analyzer (Applied Biosystems, Waltham, MA, USA). Allele sizes were scored using Geneious Prime software version 2025.2.2; https://www.geneious.com.

### 5.3. Data Analysis

MICRO-CHECKER software [29] was used to detect scoring errors and the presence of null alleles. To minimize genotyping errors, genotyping was performed independently by two researchers, and discrepancies were resolved by consensus. Individuals were assigned to clonal lineages based on identical MLGs. Missing data were excluded from comparisons; thus, individuals were considered clonal if all available alleles matched, even if one or two loci were missing. The spatial distribution of clonal groups was assessed using GPS coordinates, collected during sampling. Clonal group distribution for each site is shown in Figure S1. Genalex v. 6.51b2 [30,31,32] was used to identify identical MLGs; calculate genetic diversity parameters (number of different alleles (*Na*), the number of effective alleles (*Ae*), observed heterozygosity (*Ho*), and expected heterozygosity (*He*); perform analysis of molecular variance (AMOVA); and visualize genetic relationships using principal coordinate analysis (*PCoA*). The number of migrants per generation (*Nm)* was inferred from population differentiation assuming equal population sizes, symmetrical migration, neutrality of loci and random mating. FSTAT software [33] was used to calculate allelic richness (*Ar*) and the inbreeding coefficient (*F_IS_*). Deviations between observed and expected heterozygosity were evaluated using *F_IS_* with significance assessed by permutation tests. Effective population size (*Ne*) was estimated using NeEstimator [34] based on the linkage disequilibrium method [35], with 95% confidence intervals calculated parametrically. Latent genetic potential (*LGP*) was calculated as the difference between the total and effective number of alleles summed across loci [36]. Differences in genetic parameters between mature and juvenile cohorts were tested using Welch’s *t*-test implemented in SAS software (Version 9.4, Cary, NC, USA, 2012). Genalex was also used to calculate population differentiation, (*F_ST_* and *G*″*_ST_*) with correction for bias due to the small number of populations [37]). Bayesian clustering analysis was performed using STRUCTURE v. 2.3.4 [38,39,40,41], with the number of clusters (K) ranging from 1 to 24 and 100 replicate runs per K. Each run included 100,000 burn-in iterations, followed by 100,000 MCMC iterations. The optimal number of clusters was determined using the *ΔK* method [18] in STRUCTURE HARVESTER [42] and the results were visualized using CLUMPAK [43].

## Figures and Tables

**Table 1:** Genetic diversity parameters calculated for *Tilia cordata* Mill. populations and for different generations. For genetic conservation unit (GCU) codes, please see Table 2; the letters M and J next to the GCU code designate mature and juvenile generations, respectively. N—number of multi-locus genotypes analyzed; *Na*—number of alleles; *Ae*—effective number of alleles; *Ar*—allelic richness, based on 33 diploid individuals and all 14 loci; *Ho*—observed heterozygosity; *He*—expected heterozygosity; *F_IS_*—inbreeding coefficient; *Ne*—effective population size given with 95% confidence interval; *LGP*—latent genetic potential, calculated as multi-locus average.

Population Generation	N	*Na* ± SE	*Ae* ± SE	*Ar* ± SE	*Ho* ± SE	*He* ± SE	No. of Rare Alleles	No. of Private Alleles	*F_IS_* ± SE	*Ne* (95% CI)	*LGP* ± SE
ANK-M	41	7.5 ± 1.22	4.27 ± 0.91	7.30 ± 1.15	0.62 ± 0.08	0.63 ± 0.07	46	3	0.22 ± 0.08	33.5 (27.6–41.7)	3.24 ± 0.55
ANK-J	74	8.5 ± 1.49	4.32 ± 1.02	7.51 ± 1.24	0.62 ± 0.07	0.61 ± 0.07	65	2	−0.001 ± 0.04	146.3 (110.2–210.5)	4.18 ± 0.58
JU1-M	79	8.2 ± 1.36	4.06 ± 0.81	7.29 ± 1.15	0.60 ± 0.07	0.62 ± 0.07	52	1	0.042 ± 0.05	1740.9 (380.0–∞)	4.15 ± 0.71
JU1-J	81	7.2 ± 1.00	3.63 ± 0.58	6.54 ± 0.87	0.58 ± 0.08	0.61 ± 0.07	44	0	0.055 ± 0.08	374.0 (194.8–2438.6)	3.58 ± 0.59
JU2-M	73	8.3 ± 1.41	3.67 ± 0.65	7.30 ± 1.18	0.56 ± 0.07	0.61 ± 0.07	62	2	0.086 ± 0.06	248.9 (154.2–580.8)	4.62 ± 0.87
JU2-J	92	9.0 ± 1.80	3.76 ± 0.86	7.51 ± 1.35	0.52 ± 0.08	0.59 ± 0.07	74	9	0.130 ± 0.08	230.1 (166.1–360.3)	5.24 ± 1.07
RAS-M	91	8.1 ± 1.39	3.88 ± 0.76	7.10 ± 1.12	0.60 ± 0.08	0.60 ± 0.07	54	0	0.014 ± 0.05	315.3 (202.4–657.3)	4.26 ± 0.74
RAS-J	87	8.8 ± 1.56	3.96 ± 0.73	7.43 ± 1.24	0.60 ± 0.08	0.62 ± 0.07	66	0	0.042 ± 0.07	587.9 (306.8–4181.0)	4.83 ± 0.91
ROK-M	95	7.9 ± 1.29	4.02 ± 0.86	6.88 ± 1.06	0.54 ± 0.07	0.62 ± 0.06	51	0	0.145 ± 0.06	143.6 (112.9–192.2)	3.91 ± 0.59
ROK-J	95	7.8 ± 1.27	3.88 ± 0.71	6.69 ± 0.99	0.54 ± 0.07	0.63 ± 0.07	50	1	0.148 ± 0.07	189.4 (140.9–278.8)	3.91 ± 0.69
UKM-M	80	7.9 ± 1.37	4.35 ± 0.93	7.03 ± 1.16	0.60 ± 0.07	0.65 ± 0.07	48	1	0.070 ± 0.07	143.8 (107.2–210.4)	3.58 ± 0.65
UKM-J	91	8.1 ± 1.47	4.32 ± 1.08	7.01 ± 1.24	0.58 ± 0.08	0.63 ± 0.07	55	1	0.089 ± 0.09	470.8 (249.9–2660.6)	3.82 ± 0.61
Average	81.58	8.1 ± 0.39	4.01 ± 0.23	7.13 ± 0.33	0.60 ± 0.02	0.62 ± 0.02	113 *	1.67	0.066 ± 0.02	-	-

* The number given is an actual rare allele number when all populations are pooled together.

**Table 2:** Characteristics of *Tilia cordata* Mill. Genetic conservation units. Information is based on State Forest Service and State Forest Cadastre data as of 1st March 2026.

Site	GCUs			No. of Samples, Total (Mature/Juvenile)	Provenance Region	Average Age (y) *	Tree Species Composition (%) **
	Type	Size, (ha)	Coordinates				
ANK	Seed stand	1.84	55°32′12.0228″ N, 24°53′ 30.1014″ E	193 (93/100)	3	79	60T30P10B
JU1	Genetic reserve	3.8	55°10′5.7714″ N, 23°19′45.7134″ E	168 (83/85)	1	138	60T20S10Q10A
JU2	Genetic reserve	5.32	55°9′28.6272″ N, 23°19′32.16″ E	176 (76/100)	1	93	70T20F10S
RAS	Seed stand	7.23	55°20′25.029″ N, 23°38′48.1662″ E	195 (95/100)	1	98	40T20Q20B20S
ROK	Genetic reserve	2.87	55°47′56.331″ N, 25°48′24.8178″ E	195 (95/100)	3	84	70T10B20S
UKM	Genetic reserve	20.23	54°58′5.9118″ N, 25°11′ 8.7102″ E	182 (82/100)	3	94	50T40P10Q

* Average age (years) of mature *T. cordata* trees; ** T: *Tilia cordata*; Q: *Quercus robur*; B: *Betula pendula*; S: *Picea abies*; P: *Populus tremula*; A: *Acer platanoides*; F: *Fraxinus excelsior*. In each GCU, tree species composition is based on the tree volume.

## Data Availability

The SSR genotype data matrix is available as Supplementary Table S3.

## Supplementary Materials

The following supporting information can be downloaded at: https://www.mdpi.com/article/10.3390/plants15081207/s1.

## Abbreviations

The following abbreviations are used in this manuscript:

AMOVA	Analysis of Molecular Variance
GCU	Genetic Conservation Unit
LGP	Latent Genetic Potential
MLG	Multi-locus Genotype
PCoA	Principal Coordinate Analysis
PCR	Polymerase Chain Reaction
SFE	State Forest Enterprise
