# Supplementary material for: Genetic Diversity and Clonal Structure of Small-Leaved Lime (Tilia cordata Mill.) in Lithuanian Protected Forest Areas
Source: Plants (Basel). 2026 Apr 15;15(8):1207. doi: 10.3390/plants15081207 (PMC13119874; doi:10.3390/plants15081207)

Genetic Diversity and Clonal Structure of Small-leaved Lime (*Tilia cordata* Mill.) in Lithuanian Protected Forest Areas

Rita Verbylaitė [1*], Jūratė Lynikienė [1], Artūras Gedminas [1], Valeriia Mishcherikova [1] Virgilijus Baliuckas [1] and Vytautas Suchockas [1]

Figure S1. Sampling maps and clonal groups of investigated GCUs. Clonal groups are marked by drop shape, while single copy genotypes are marked by dots. Different clonal groups are marked by different colors. Mature trees are marked by red dots, while juveniles – yellow dots.

A Anykščiai ANK seed stand

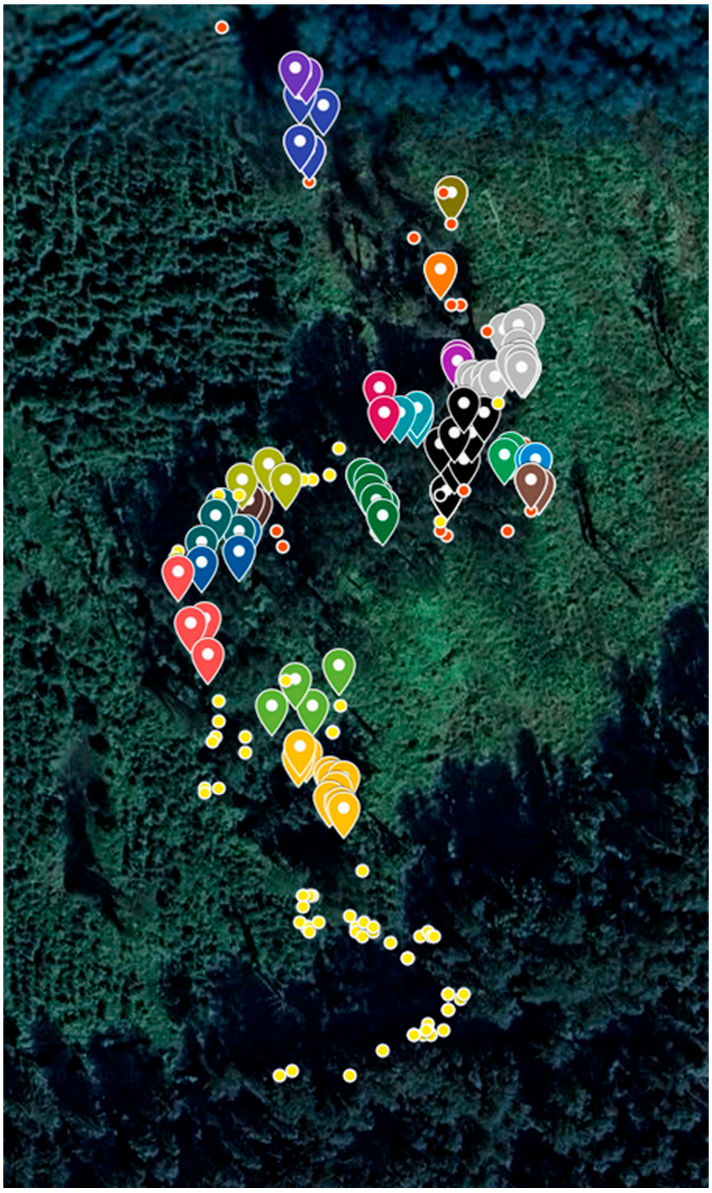

B Jurbarkas 1 JU1 genetic reserve

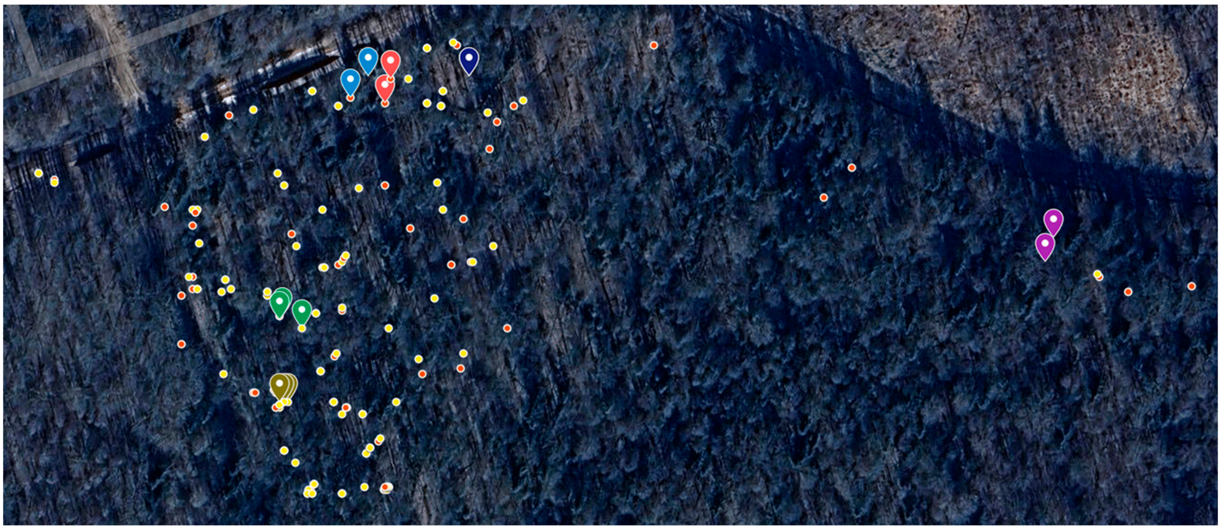

C Jurbarkas 2 JU2 genetic reserve

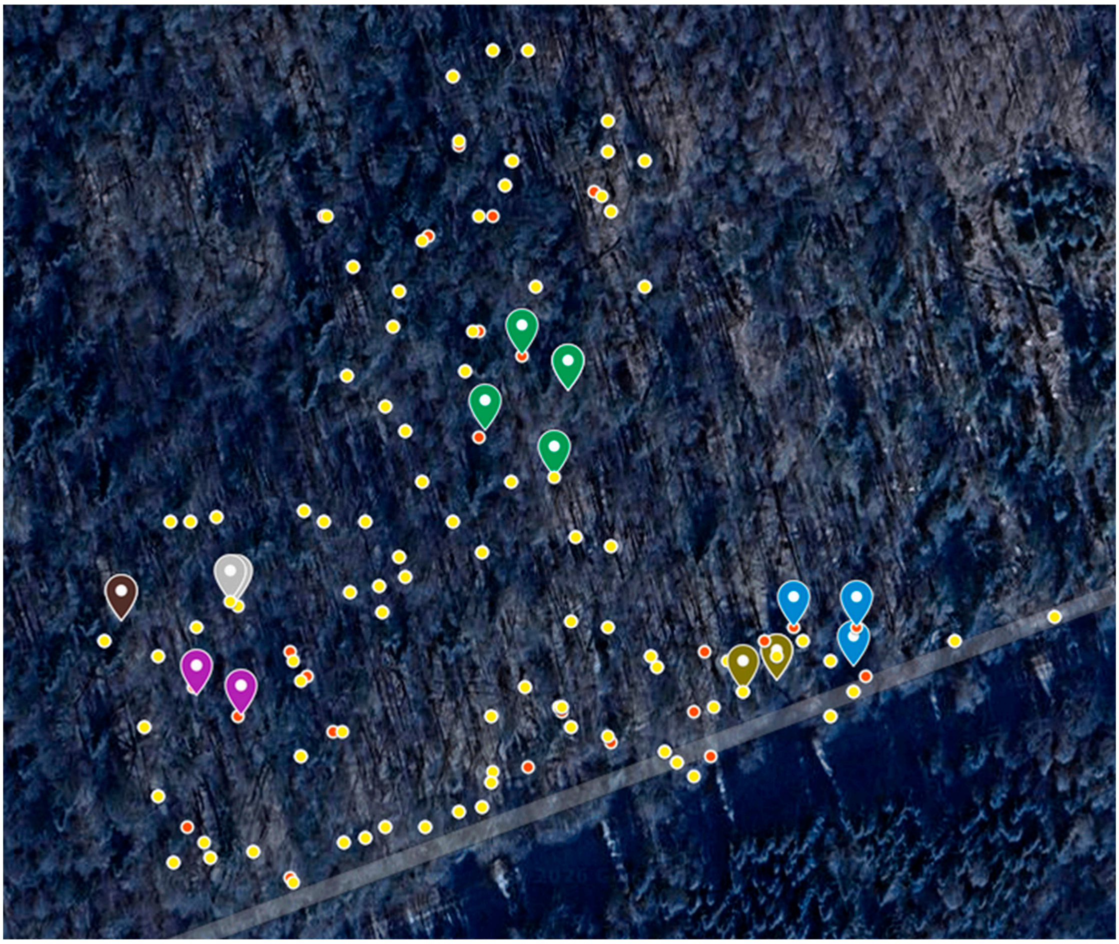

D Raseiniai RAS seed stand

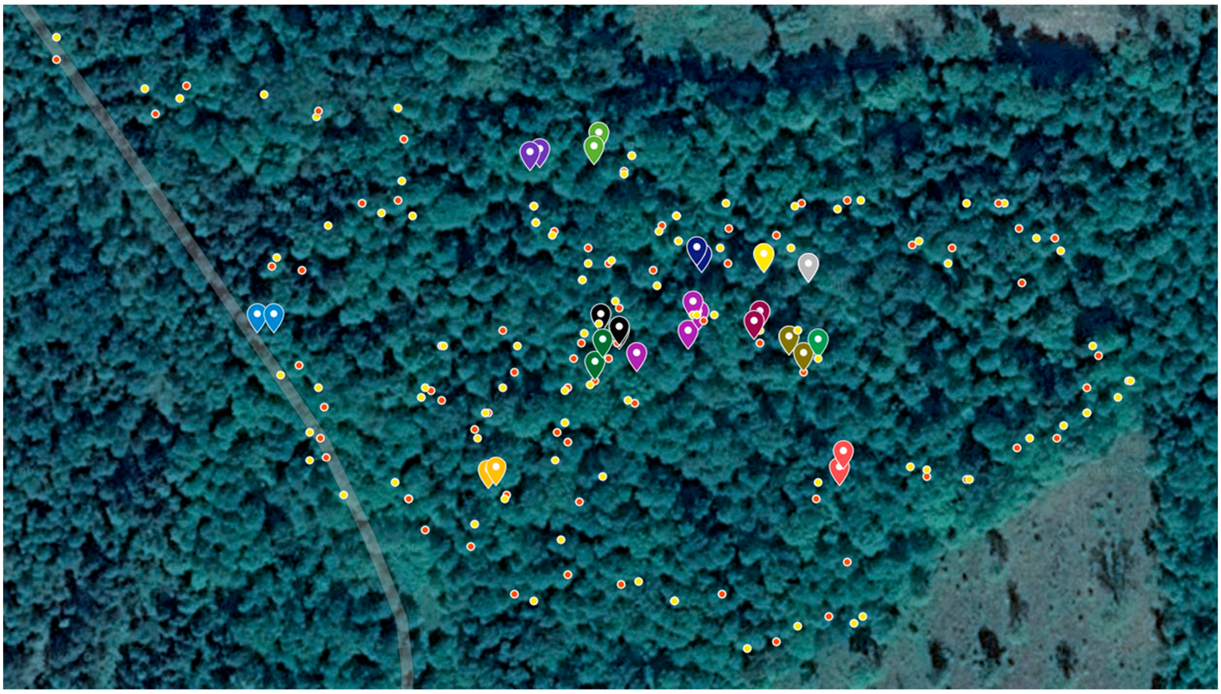

E Rokiškis ROK genetic reserve

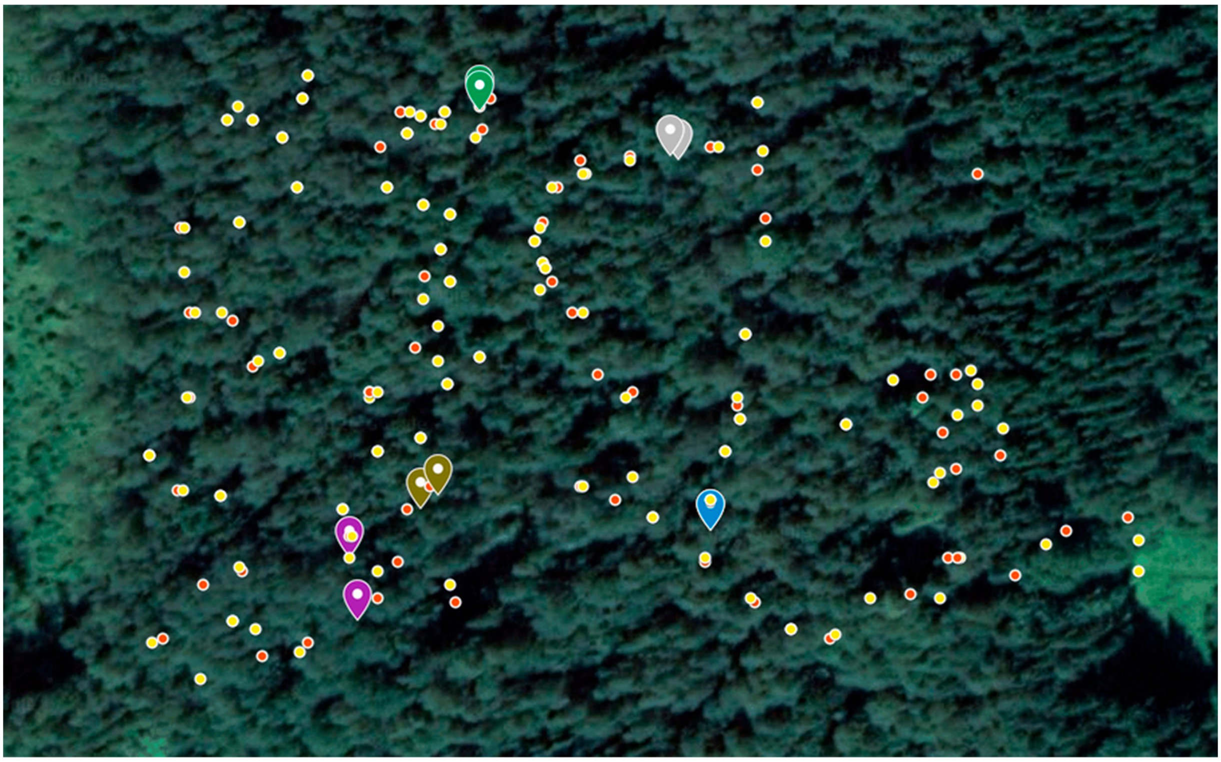

F Ukmergė UKM genetic reserve

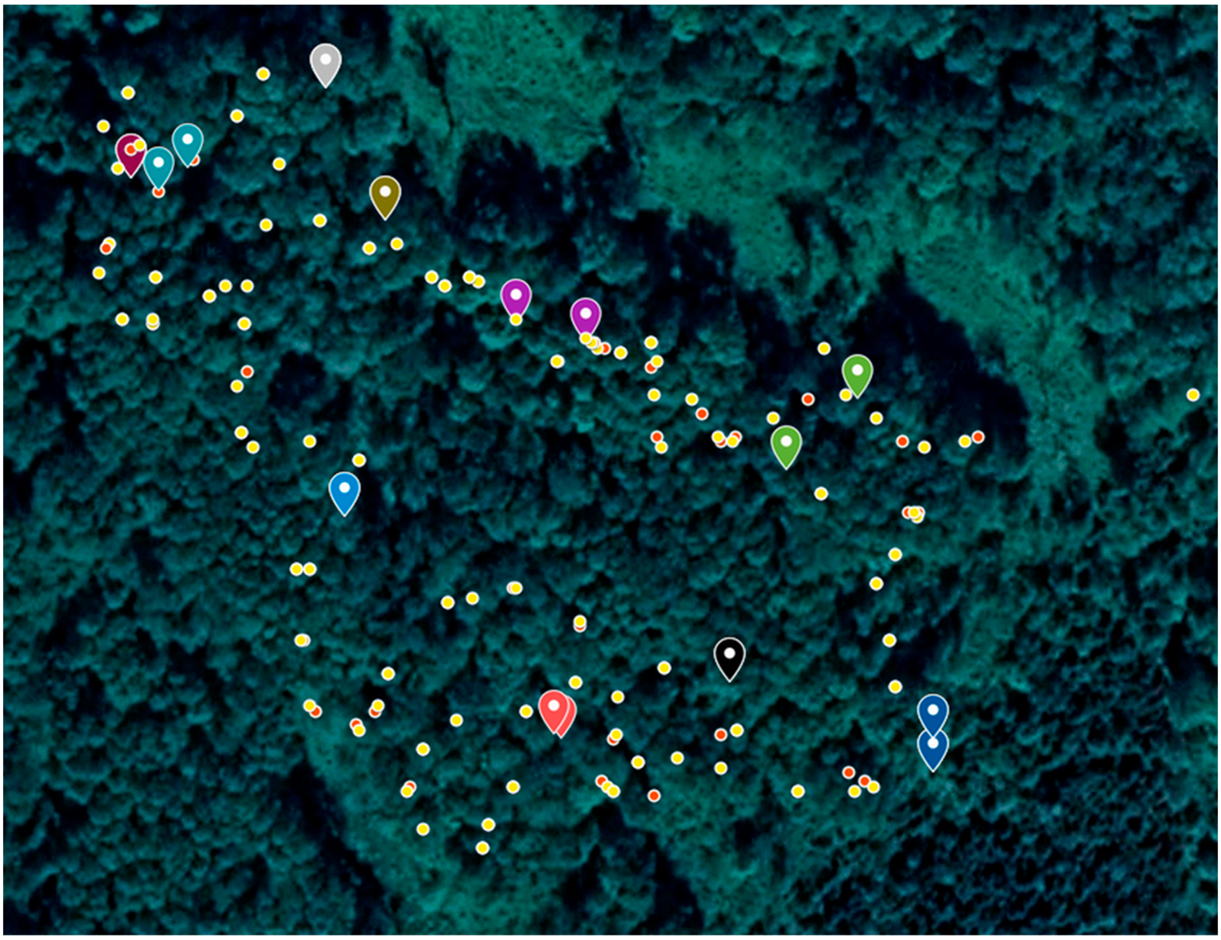

Supplement: Supplementary file 1 [file plants-15-01207-s001.zip › plants-4239384-Figure S1.pdf]
