# Supplementary material for: Genetic Diversity and Clonal Structure of Small-Leaved Lime (Tilia cordata Mill.) in Lithuanian Protected Forest Areas
Source: Plants (Basel). 2026 Apr 15;15(8):1207. doi: 10.3390/plants15081207 (PMC13119874; doi:10.3390/plants15081207)
Supplement: Supplementary file 1 [file plants-15-01207-s001.zip › plants-4239384-Table S2.pdf]

Genetic Diversity and Clonal Structure of Small-leaved Lime (*Tilia cordata* Mill.) in Lithuanian Protected Forest Areas

Rita Verbylaitė [1*], Jūratė Lynikienė [1], Artūras Gedminas [1], Valeriia Mishcherikova [1] Virgilijus Baliuckas [1] and Vytautas Suchockas [1]

Table S2. The probability of likelihood for the number of clusters for *Tilia cordata,* as determined by the ΔK criterion [33].

K	Reps	Mean LnP(K)	Stdev LnP(K)	Ln'(K)	|Ln''(K)|	Delta K
1	100	-37852.9840	0.3572	NA	NA	NA
2	100	-37559.5810	7.6550	293.403000	82.030000	10.715918
3	100	-37348.2080	18.9938	211.373000	57.065000	3.004401
4	100	-37193.9000	27.1956	154.308000	98.327000	3.615555
5	100	-37137.9190	52.1799	55.981000	3.072000	0.058873
6	100	-37085.0100	70.9320	52.909000	66.727000	0.940718
7	100	-36965.3740	57.5953	119.636000	60.172000	1.044737
8	100	-36905.9100	66.3765	59.464000	57.341000	0.863875
9	100	-36903.7870	384.0546	2.123000	159.584000	0.415524
10	100	-36742.0800	506.6607	161.707000	131.123000	0.258798
11	100	-36711.4960	272.0717	30.584000	7.669000	0.028187
12	100	-36673.2430	416.9717	38.253000	64.788000	0.155377
13	100	-36570.2020	125.4010	103.041000	152.054000	1.212542
14	100	-36619.2150	388.4675	-49.013000	80.289000	0.206681
15	100	-36587.9390	477.1012	31.276000	10.417000	0.021834
16	100	-36567.0800	513.4867	20.859000	67.677000	0.131799
17	100	-36478.5440	129.1824	88.536000	237.157000	1.835830
18	100	-36627.1650	569.5231	-148.621000	152.247000	0.267324
19	100	-36623.5390	487.9474	3.626000	34.264000	0.070221
20	100	-36585.6490	558.2371	37.890000	134.859000	0.241580
21	100	-36682.6180	605.0582	-96.969000	78.012253	0.128933
22	99	-36701.5747	813.4209	-18.956747	118.271505	0.145400
23	100	-36838.8030	599.3331	-137.228253	160.467886	0.267744
24	101	-36815.5634	500.4247	23.239634	NA	NA
